# Association of autoantibody levels with different stages of age-related macular degeneration (AMD): Results from the population-based Gutenberg Health Study (GHS)

**DOI:** 10.1007/s00417-023-06085-2

**Published:** 2023-05-09

**Authors:** Christina A. Korb, Karl J. Lackner, Dominik Wolters, Alexander K. Schuster, Stefan Nickels, Vanessa M. Beutgen, Thomas Münzel, Philipp S. Wild, Manfred E. Beutel, Irene Schmidtmann, Norbert Pfeiffer, Franz H. Grus

**Affiliations:** 1 Department of Ophthalmology, University Medical Center of the Johannes Gutenberg-University Mainz, Langenbeckstr. 1, 55131 Mainz, Germany; 2 Institute for Clinical Chemistry and Laboratory Medicine, University Medical Center of the Johannes Gutenberg-University Mainz, Mainz, Germany; 3 Center for Cardiology, University Medical Center of the Johannes Gutenberg-University Mainz, Mainz, Germany; 4 Preventive Cardiology and Preventive Medicine, Department of Cardiology, University Medical Center of the Johannes Gutenberg-University Mainz, Mainz, Germany; 5 Center for Thrombosis and Hemostasis, University Medical Center of the Johannes Gutenberg-University Mainz, Mainz, Germany; 6 German Center for Cardiovascular Research (DZHK), partner site Rhine Main, Mainz, Germany; 7 Institute of Molecular Biology (IMB), Mainz, Germany; 8 Department of Psychosomatic Medicine and Psychotherapy, University Medical Center of the Johannes Gutenberg-University Mainz, Mainz, Germany; 9 Institute of Medical Biostatistics, Epidemiology and Informatics, University Medical Center of the Johannes Gutenberg-University Mainz, Mainz, Germany

**Keywords:** Age-related macular degeneration, autoantibodies, antigen-microarrays, Gutenberg Health Study

## Abstract

**Purpose:**

Anti-retinal autoantibodies are assumed to be associated with age-related macular degeneration (AMD). To our knowledge, this is the first evaluation of autoantibodies in human sera of participants with different stages of AMD in a large population-based, observational cohort study in Germany.

**Methods:**

The Gutenberg Health Study (GHS) is a population-based, observational cohort study in Germany, including 15,010 participants aged between 35 and 74. Amongst others, non-mydriatic fundus photography (Visucam PRO NM™, Carl Zeiss Meditec AG, Jena, Germany) was performed. Fundus images of the first 5000 participants were graded based on the Rotterdam Eye Study classification. Sera of participants with AMD (*n*=541) and sera of age-matched participants without AMD (*n*=490) were analyzed by antigen-microarrays. Besides descriptive statistics, autoantibody-levels were compared by Mann-Whitney-U test and the associations of level of autoantibodies with AMD were calculated by logistic regression analysis. Likewise, possible associations of the autoantibodies and both clinical and laboratory parameters on AMD subjects were analyzed.

**Results:**

Autoantibodies against transferrin (*p*<0.001) were significantly downregulated in participants with early AMD and soft, distinct drusen (≥63 μm) or pigmentary abnormalities only compared to Controls. Mitogen-activated protein kinase 3 (*p*=0.041), glutathione peroxidase 4 (*p*=0.048), clusterin (*p*=0.045), lysozyme (*p*=0.19), protein kinase C substrate 80K-H (*p*=0.02), heat shock 70 kDa protein 1A (*p*=0.04) and insulin (*p*=0.018) show a trend between Control and participants with early AMD and soft, distinct drusen (≥63 μm) or pigmentary abnormalities only.

**Conclusions:**

This study contributes to a growing knowledge of autoantibodies in association with different AMD stages compared to controls in the context of a large population-based study in Germany. Especially autoantibodies against inflammatory proteins were downregulated in participants with early AMD and soft, distinct drusen (≥63 μm) or pigmentary abnormalities only. 
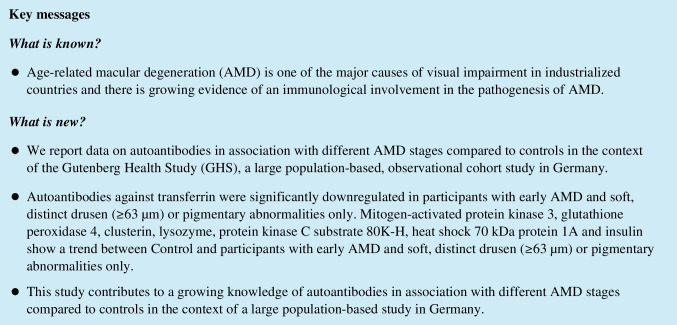

**Supplementary Information:**

The online version contains supplementary material available at 10.1007/s00417-023-06085-2.

## Introduction

Age-related macular degeneration (AMD) is one of the major causes of irreversible visual impairment in industrialized countries [1].

In a previous study the prevalence of AMD in the Gutenberg Health Study (GHS) cohort was determined on the basis of fundus photographs from 4,340 participants [2]. Aging is the greatest risk factor for AMD, accordingly, in a previous study the prevalence of AMD in the cohort increased with age; however, although AMD is usually defined to occur after age 50, signs of early AMD were found in 3.8 % of individuals in the youngest age group (younger than 45 years) [2]. Additionally, several studies during the last years reported the involvement of anti-retinal autoantibodies in ocular disorders, such as retinal vasculitis [3], retinopathy [4, 5], retinitis pigmentosa [6] and also AMD [7–12]. These studies support the growing evidence of an immunological involvement in the pathogenesis of AMD. Accordingly, in a previous study we could demonstrate a difference in Immunoglobulin G (IgG) antibody patterns against retinal antigens between patients with wet AMD and healthy volunteers [13]. Until now the relevance and the role of autoantibodies in the pathogenesis of AMD is unknown and it is unclear if antibodies play a causative role during the pathogenesis of AMD or might appear as secondary effects during the disease’s progression. Autoantibody evaluation in serum may provide a minimally invasive procedure with impact for prediction of disease activity, prognosis and especially response to treatment in the future.

The aim of this study is to provide different serological autoantibody biomarkers in relation to different stages of AMD in comparison to healthy control subjects.

We therefore evaluated the presence of different serologic autoantibody levels in study participants with AMD in the population-based Gutenberg Health Study (GHS).

## Materials and methods

### Study population

The Gutenberg Health Study (GHS) is a population-based, prospective, single-center cohort study at the medical center of the Johannes Gutenberg University Mainz in Germany [14]. The sample was randomly drawn via local residents’ registration offices and equally stratified by sex for each decade of age. Exclusion criteria for participation in the GHS were physical or mental inability or insufficient knowledge of German to participate in the examinations in the study center. The baseline examination with a total of 15,010 participants aged 35 to 74 years was conducted from 2007 to 2012. We included a subsample of 541 subjects with AMD and 490 control subjects in this analysis.

The GHS mainly focuses on the general health status of the population to develop better cardiovascular risk stratification. Thus, the ophthalmologic examination is embedded in an interdisciplinary setting including general examinations emphasising cardiovascular parameters, completed by psychological questionnaires, and laboratory tests with biobanking for proteomic and genetic analyses. The setting of our study enables the detection of associations between ophthalmic and systemic findings and their development over time [2].

The ophthalmic part has been described in detail elsewhere [15]. In brief, we conducted measurements of objective refraction and distance-corrected visual acuity, intraocular pressure, visual field testing, pachy- and keratometry, and posterior segment photography.

### AMD grading

The fundus images were taken with a non-mydriatic fundus camera (Visucam PRO NMTM, Carl Zeiss AG, Jena, Germany) in a darkened room and with the pupil’s natural width. Three photographs were taken of each eye: at 30° and 45° centered on the optic nerve, and at 30° centered on the macula.

We evaluated data from the first 5,000 subjects enrolled in the GHS between April 2007 and October 2008. The grading was performed based on the Rotterdam Eye Study classification and was described in detail previously [2, 16]. As control, a group of age- and sex-matched participants without AMD was used. The control group was defined as having no signs of AMD at all, without drusen or pigmentary abnormalities in either eye. AMD1 was defined as early AMD (stage 1a: soft, distinct drusen (≥63 μm) only; stage 1b: pigmentary abnormalities only, no soft drusen (≥63 μm), AMD2 was defined as early AMD (stage 2a: soft, indistinct drusen (≥125 μm) or reticular drusen only; stage 2b: soft, distinct drusen (≥63 μm) with pigmentary abnormalities), AMD3 was defined as early AMD (stage 3: soft, indistinct (≥125 μm) or reticular drusen with pigmentary abnormalities) and AMD4 was defined as late AMD in one eye corresponding to geographic atrophy or neovascular AMD [16].

AMD4 participants were defined as atrophic or neovascular AMD. Participants with maculopathy unrelated to AMD were excluded. When different stages of AMD were noted between the right and the left eye, we chose the classification for the worse eye. Cases of strict unilateral, nonspecific maculopathy were grade as being unrelated to AMD.

### Sera

Full blood samples of the GHS participants with different AMD stages and matched participants without AMD were allowed to clog for 30 minutes and were centrifuged for 10 minutes at 4°C and 1000g. The supernatant was collected and serum samples were stored at -80°C until further use.

### Microarray manufacturing and incubation

Customized microarrays were manufactured in our lab using a non-contact piezo-dispenser (SciFEXARRAYER S3, Scienion, Berlin, Germany). The 61 selected antigens (S1 Table) were spotted in triplicates onto nitrocellulose coated microarray slides (AVID Oncyte, 16 Pad NC slides, Grace Biolabs, Bend, Oregon, USA). The 61 antigens were selected based on literature and previous studies regarding AMD and (eye-) diseases with autoimmune components. The spotting process was carried out in a humidity chamber with humidity set to 60%. To allow optimal immobilization of the proteins on the microarray surface, slides were kept on the spotter platform to dry overnight prior to incubation. For the incubation steps, the slides were clamped into incubation chambers (ProPlate Multiwell chambers, Grace Biolabs, Bend, USA), separating the slide into 16 distinct subarrays. The following incubation steps were performed at 4°C on an orbital shaker. To decrease background signals, the arrays were incubated with blocking buffer (Super G, Grace Biolabs, Bend, Oregon, USA) for one hour. Afterwards, the blocking buffer was removed and residual buffer was removed by washing three times with phosphate-buffered saline containing 0.5% Tween-20 (PBST). The arrays then were incubated with 100μL serum samples in a 1:250 dilution in PBS overnight. Positive and negative controls were included on each slide. Slides were washed again three times with PBST followed by incubation for one hour with a secondary anti-human antibody conjugated with Alexa fluor 647 (Alexa Fluor® 647 AffiniPure Goat Anti-Human IgG, Fcγ fragment specific, 109-605-008, Jackson Immunoresearch) in a 1:500 dilution in PBS. After the incubation with the secondary antibody, the slides were washed two times with PBST and two times with ultrapure water. Slides were dried for two minutes in a vacuum concentrator (SpeedVac, Thermo Scientific, Waltham, Massachusetts, USA).

### Image acquisition and data preprocessing

The microarray slides were scanned with a high resolution confocal laser scanner (428 Array Scanner, Affymetrix, Santa Clara, California, USA). The scans were saved as 16 bit Tagged Image File Format (TIFF)images. Spot intensities were quantified with an image processing software (Imagene 5.5, BioDiscovery Inc., Los Angeles, California, USA). Spots with poor quality were manually flagged and removed from further analysis. Local background intensity was subtracted from median pixel intensities and the means from the three technical replicates were calculated. The resulting data for each array was normalized using a constant scale factor.

### Statistical analysis

Descriptive statistics included for dichotomous parameters absolute and relative frequencies, for continuous measurements mean and standard deviations for approximately normal distributed data, and otherwise median and interquartile ranges. To find possible differences between subjects with AMD and controls in the underlying antibody data Mann-Whitney’s U test was used. Shapiro Wilk’s test was computed to test normal distribution of autoantibody levels.

In a second step, the three different groups (Control, AMD1 and AMD2) were compared using Kruskal-Wallis-Test followed by between-groups comparisons with Bonferroni correction.

Logistic regression analysis was performed with AMD Group (yes/no) as dependent variable and Antibody-level (per 1000 units) as independent variable adjusted for smoking, body-mass-index, glycated hemoglobin (HbA1c) level, level of high-density lipoproteins, of low-density lipoproteins and triglycerides.

Protein interaction networks, biological pathways and protein clusters were generated with Ingenuity Pathway Analysis (IPA v.01–04).

The statistical analysis was performed in Statistica (Statistica 13, Statsoft, Tulsa, Oklahoma, USA) and in R version 3.3.1 (R Core Team (2016). R: A language and environment for statistical computing. R Foundation for Statistical Computing, Vienna, Austria. URL https://www.R-project.org/.).

## Results

In this nested case-control study, 1116 subjects (601 AMD patients and 515 control subjects) were included. 85 subjects had less than 75% valid values for the antibodies and were therefore excluded. Finally, 541 subjects with AMD (AMD1: *n*=454, AMD2: *n*=64, AMD3: *n*=15, AMD4: *n*=8) and 490 control subjects were included. Characteristics of the study population are demonstrated in Tables 1 and 2.

**Table 1:** Characteristics of the study sample. Characteristics of the study sample from the Gutenberg Health Study regarding age and sex

Group	N	Mean Age	SD	Interquartile range	% of population	Male	Female
Control	490	60.2	9.5	53-68	47.5	258	232
AMD 1	454	60.0	9.6	53-68	44.0	249	205
AMD 2	64	66.7	7.0	63-72	6.2	30	34
AMD 3	15	65.3	8.7	63-71	1.6	7	8
AMD 4	8	67.0	8.8	67-72	0.8	4	4
AMD (1-4)	541	61.0	9.6	54-69	52.5	290	251

**Table 2:** Characteristics of the study sample. Characteristics of the study sample from the Gutenberg Health Study regarding age, BMI, level of homocysteine, Hba1c, fasting glucose level, HDL-C, LDL-C and triglyceride

Parameter	Control		AMD (AMD 1-4)	
	Median	Interquartile range	Median	Interquartile range
Age	60.2	53-68	61.0	54-69
Sex [female]	47.5%		46.4%	
Smoking [yes]	14.3%		16.3%	
BMI [kg/m^2^]	26.7	24.6 - 29.7	26.9	24.4 - 29.4
level of homocysteine [mg/dl]	10.8	9.2 - 13.0	10.8	9.1 - 12.9
HbA1C [%]	5.4	5.1 - 5.9	5.5	5.2 - 5.9
fasting glucose level [mg/dl]	94.0	87.0 - 154.0	94.0	87.0 - 101.0
HDL-C [mg/dl]	55.0	45.0 - 66.0	55.0	45.0 - 67.0
LDL-C [mg/dl]	145.0	119.0 - 167.0	142.0	119.0 - 167.0
Triglycerides [mg/dl]	112.5	84.0 - 154.0	106.0	81.0 - 144.0

Shapiro Wilk’s test showed that neither the laboratory parameters nor the antibody levels were normally distributed. Therefore, Mann-Whitney’s U test was applied for the following analysis.

Antibody levels were compared between subjects with and without AMD. 8 out of 61 antibodies showed different levels between both groups (*p*<0.05): a decrease of autoantibody levels against mitogen-activated protein kinase 3 (*p*=0.016), glutathione peroxidase 4 (*p*=0.026), clusterin (*p*=0.028), lysozyme (*p*=0.029), transferrin (*p*<0.001) and protein kinase C substrate 80K-H (*p*=0.013) was observed in the AMD group (Table 3). On the other side, heat shock 70 kDa protein 1A (*p*=0.028) and insulin (*p*=0.017) were upregulated. The other autoantibodies showed no up- or downregulation.

**Table 3:** Description of the antibodies. Characteristics of the antibodies including median and interquartile range for each antibody individually for ‘Control’ and ‘AMD’ group. P-value is the result of Mann-Whitney’s U test between both groups

	Control		AMD (AMD 1-4)		
Antibody	Median	Interquartile range	Median	Interquartile range	p-value
SRP14	22055	13106 -45062	22135	12925 -43984	0.648
MAPK3	22347	14716 -33258	20220	9959 -30293	0.016
GPX4	6666	4424 -8942	6161	3634 -8428	0.026
GPD2	12874	8166 -20191	12328	7360 -19426	0.334
LPPR3	32893	18854 -52056	34506	21217 -54892	0.243
SCFD1	10408	5422 -21656	10794	6260 -22581	0.344
ACTA1	19951	13068 -28181	20243	11982 -29331	0.861
ENO2	16184	8502 -29218	17980	9181 -32167	0.092
GST	5037	2881 -9356	5511	3188 -9340	0.290
ALB	14407	8916 -23392	13876	8969 -22870	0.669
CLUS	5854	3089 -8621	6397	4057 -9280	0.028
LYZ	9473	4834 -21144	8449	4368 -14844	0.029
MBP	7803	5577 -11904	7916	5376 -12296	0.979
PKC	413	82 -1348	425	99 -1423	0.529
SOD	1451	690 -2523	1288	626 -2151	0.113
FN1	32867	20077 -43063	32907	20665 -43645	0.664
ANXA5	562	256 -991	576	263 -1077	0.331
SNCG	7219	4562 -11958	7028	4518 -10983	0.677
B_L_CRYS	4347	2331 -6469	4312	2219 -7131	0.393
USP10	4019	1635 -6061	4121	1489 -6735	0.776
GAPDH	35496	25072 -46722	35766	24691 -47052	0.800
TF	22991	14802 -30996	19674	12543 -28047	0.000
GFAP	20852	15477 -28467	21438	15454 -30105	0.539
CALR	44053	32602 -58217	45723	32825 -61939	0.441
APOA1	15633	11217 -21669	15015	10303 -22635	0.430
groEL2	27022	18079 -40714	26273	18246 -39845	0.674
TNNI3	27389	19977 -37123	27269	20150 -38569	0.553
HARS	15291	11642 -21846	15927	12318 -21828	0.370
PPIA	41318	32585 -52510	42122	31919 -54050	0.610
CA2	63095	51235 -80148	61772	48514 -77257	0.083
HSPD1	38709	23052 -55892	37122	22096 -55990	0.706
MUC5B	8167	5833 -11180	7896	6008 -10623	0.837
OGFR	6979	4740 -9358	6920	4996 -9474	0.654
TG	67546	54340 -90431	67395	53329 -81785	0.108
TNF	31799	23344 -43639	31758	20410 -44774	0.378
INS	1049	321 -5088	1354	425 -29980	0.017
SPTA1	689	359 -1317	785	416 -1355	0.176
VEGF	8565	1205 -31914	6297	1163 -31611	0.273
HSPE1	28491	15181 -43859	27337	8835 -43054	0.165
NTF3	39684	28111 -52896	39954	28988 -55664	0.476
SNCA	2894	1831 -4562	2823	1749 -4598	0.758
PDIA3	38136	27117 -51916	39113	27513 -52513	0.785
DCD	8162	5331 -12560	7992	5370 -12636	0.860
GLUL	4129	2721 -6667	4128	2695 -6619	0.939
ACO2	552	254 -1137	555	213 -1052	0.300
TOP1	48539	36171 -59593	49835	38409 -60667	0.259
NTF4	1508	693 -3140	1739	790 -3309	0.176
PRKCSH	12905	6581 -22771	11196	3988 -20334	0.013
DPYSL2	1716	723 -4071	1708	728 -4067	0.928
UCHL1	3379	1791 -5936	3474	2045 -6190	0.280
PEBP1	1314	680 -2152	1279	654 -2176	0.989
CKB	524	212 -1125	469	198 -960	0.152
GNB1	446	154 -943	453	151 -940	0.762
IGLL1	961	429 -1680	907	398 -1547	0.483
HSPB1	10221	6740 -17929	10949	6564 -17887	0.667
HSPA1A	4884	2086 -10713	5422	2601 -11390	0.028
SERPINA	7719	5274 -10678	7836	5037 -10869	0.860
TTR	883	356 -1610	905	407 -1754	0.314
SFN	45576	27015 -61543	44644	27425 -62515	0.674
EIF4A1	4314	2697 -7455	4025	2525 -7241	0.315
BDNF	25174	11130 -42511	23154	10940 -43770	0.778

Logistic regression analysis showed, that the association between AMD and autoantibody level was still present after including the following parameters as independent variables into the model to adjust for: (yes vs. no), body-mass-index, HbA1c level, level of high-density lipoproteins, of low-density lipoproteins and triglycerides (all continuous) in case of mitogen-activated protein kinase 3 (*p*=0.04), lysozyme (*p*=0.013), transferrin (*p*<0.007) and protein kinase C substrate 80K-H (*p*=0.019) and insulin (*p*=0.02) (Table 4).

**Table 4:** Result of logistic regression. Result of logistic regression between AMD-variable (dependent) and autoantibody-level (independent) adjusted for smoking, HbA1c, BMI, LDL, HDL and Triglycerides

	Crude			adjusted		
Logistic regression (per1000U)	OR	95% CI	p-value	OR	95%CI	p-value
MAPK3	0.99	0.98, 1.00	0.04	0.99	0.98, 1.00	0.039
GPX4	0.98	0.96, 1.01	0.114	0.98	0.96, 1.01	0.138
CLUS	1.01	0.99, 1.03	0.353	1.01	0.99, 1.03	0.382
LYZ	0.99	0.98, 01.00	0.013	0.99	0.98, 1.00	0.023
TF	0.99	0.98, 1.00	0.007	0.99	0.98, 1.00	0.013
INS	1.01	1.00, 1.01	0.02	1.01	1.00,1.01	0.041
PRKCSH	0.99	0.98, 1.00	0.019	0.99	0.98, 1.00	0.025
HSPA1A	1.02	1.00, 1.03	0.011	1.02	1.00, 1.03	0.012

**Fig. 1:**
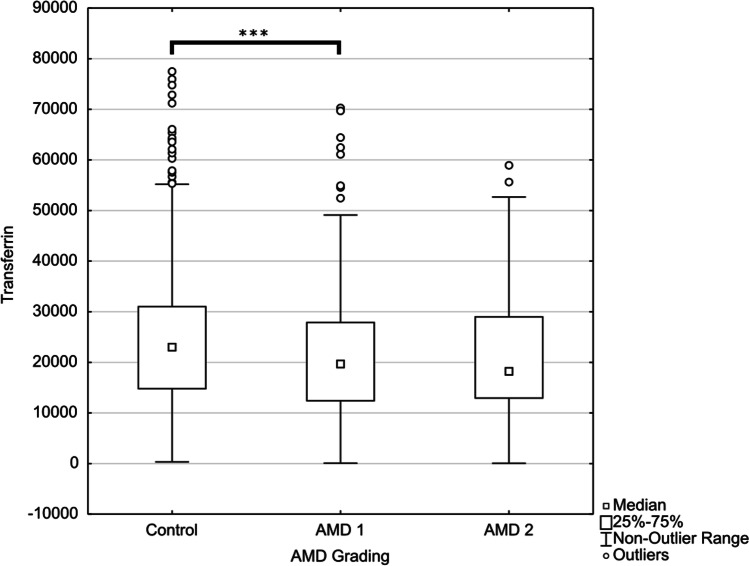
Box-and-whisker plot for detected autoantibodies. Box-and-whisker plot for detected autoantibodies. The X-axis represents the three groups (Control, AMD 1 and AMD 2) and the Y-axis the measured intensity values. Using Kruskal-Wallis-Test followed by between-groups comparisons only transferrin (*p*<0.001) could be confirmed between Control and AMD1 after applying the false discovery rate (FDR) of 5% using the Benjamini-Hochberg procedure

For further detailed investigation of the different AMD stages, Control, AMD1 and AMD2 were compared. Using Kruskal-Wallis-Test followed by between-groups comparisons only transferrin (*p*<0.001) could be confirmed between Control and AMD1 after applying the false discovery rate (FDR for *n*=183; 3 groups, 61 antibodies) of 5% using the Benjamini-Hochberg procedure. Mitogen-activated protein kinase 3 (*p*=0.041), glutathione peroxidase 4 (*p*=0.048), clusterin (*p*=0.045), lysozyme (*p*=0.19), protein kinase C substrate 80K-H (*p*=0.02), heat shock 70 kDa protein 1A (*p*=0.04) and insulin (*p*=0.018) show a trend between Control and AMD1, but are rejected as significant after applying the FDR. No significant regulations could be observed between AMD2 versus AMD1 or Control (Fig 1).

Protein interaction networks showed most of the proteins targeted by the regulated antibodies are involved in Connective Tissue Disorders, Organismal Injury and Abnormalities, Skeletal and Muscular Disorders and Neurological Diseases (Table 5). Furthermore, canonical pathways were discovered including multiple targeted proteins (Table 6).

**Table 5:** Ingenuity Pathway Analysis. Ingenuity Pathway Analysis revealing predicted involved processes or diseases

Name	Molecules
Connective Tissue Disorders	7
Organismal Injury and Abnormalities	8
Skeletal and Muscular Disorders	7
Infectious Diseases	4
Neurological Disease	7

**Table 6:** Top Canonical Pathways. Top Canonical Pathways discovered using Ingenuity Pathway Analysis

Name	Overlap
Clathrin-mediated Endocytosis Signaling	1.9 % - 4/207
Apelin Adipocyte Signaling Pathway	3.8 % - 3/80
LXR/RXR Activation	2.5 % - 3/121
FXR/RXR Activation	2.4 % - 3/126
IL-12 Signaling and Production in Macrophages	2.1 % - 3/146

## Discussion

In our study, we report an evaluation of autoantibodies in human sera by antigen-microarrays of participants with different stages of AMD and healthy controls in a large population-based, prospective, observational cohort study in Germany (The Gutenberg Health Study (GHS)). Autoantibodies against mitogen-activated protein kinase 3, glutathione peroxidase 4, clusterin, lysozyme, transferrin and protein kinase C substrate 80K-H were downregulated in AMD 1 compared to Control. Autoantibodies against transferrin were significantly downregulated in participants with early AMD and soft, distinct drusen (≥63 μm) or pigmentary abnormalities only compared to Controls. Autoantibodies against heat shock 70kDa protein 1A and insulin were upregulated in AMD 1.

Transferrin might serve as a component of oxidative defense system of the retina [17]. Chowers and coworkers demonstrated that transferrin expression is increased in the retinas of patients with AMD relative to those of healthy control patients supposed a role in the oxidative defense system [17]. Transferrin is an iron transport protein and is expressed at high levels in the retina [17]. Wysokinski et al. showed that the serum level of transferrin was higher in the AMD group (*p*< 0.001) than in the control, but the total serum iron levels did not differ between both groups [18]. Also, genetic polymorphisms in iron homeostasis genes constituted potential risk markers for AMD [18]. In our study, autoantibodies against transferrin were significantly downregulated in the AMD 1 group compared to individuals without AMD.

Yang and coworkers demonstrated, that inhibition of protein kinase C increased retinal pigment epithelium (RPE) cell susceptibility to complement attack [19]. Complement activation is clearly involved in the pathogenesis of AMD [20, 21]. Zhang et al. also suggested, that light induced phagocytic crisis of RPE cells may result from the down-regulation of protein kinase C alpha (PKC-α)/ezrin signaling, with PKC-α/ezrin as a key signal pathway regulating the phagocytosis of many types of cells [22, 23]. Accordingly, autoantibodies against protein kinase C substrate 80K-H were downregulated in AMD 1 in our study.

The etiology of AMD is complex and multifactorial, but there is evidence that oxidative stress is involved in AMD pathogenesis and progression [24, 25]. In recent years, studies indicated that MAPK (mitogen-activated protein kinases) pathways are involved in the development of AMD [25, 26]. Oxidative stress and reactive oxygen species (ROS) have been implicated in the activation of various signaling pathways, including the MAPKs [25, 27]. Several studies suggested that MAPKs play a major role in oxidative stress-induced RPE degeneration [25, 28]. Koinzer and coworkers showed a biphasic activation of MAPK3 ERK1/2 after oxidative insult in RPE cells, of which the late activation was pro-apoptotic [28]. Recently, there has been described an important role of ERK1/2 activation in the pathogenesis of geographic atrophy, a late manifestation of AMD [29]. Autoantibodies against MAPK3 were downregulated in AMD 1 compared to Control in our analysis. Hypothetically, the downregulation in the AMD 1 group might indicate a loss of the protective function and an enhancement of the pro-apoptotic activation with resulting RPE degeneration.

Anti-carbonic anhydrase II (anti-CA II) autoantibodies were detected in sera of patients with autoimmune retinopathies (AR), including cancer-associated retinopathy (CAR) and also in normal population [30]. Anti-Carbonic anhydrase II is involved in retinal disorders [31, 32], additionally, antiretinal antibodies against CAII have been detected in the sera of patients with autoimmune diseases, such as type 1 diabetes [33], Sjögren´s syndrome [34] or systemic lupus erythematodes [35]. Because of the wide occurrence of anti-CAII antibodies in diseases, it is likely that all of these diseases may have a common pathogenic mechanism based on autoimmune responses [30]. Transferably, suspected immunologic origins of AMD include e.g. the alternative complement pathway, immune cell activation, and autoimmunity [36].

Basic proteins such as lysozyme were shown to promote melanin polymerization under acidic conditions [37]. In the present study, autoantibodies against lysozyme were downregulated in AMD 1 compared to Control. Sarangajan et al. stated that the ability of the melanin polymer to act as an anti- or as an pro-oxidant may be dependent upon its state of aggregation [37]. Age related changes in the state of aggregation, as e.g. also in age-related macular degeneration, are suspected to alter the degree of polimerization and also the pro-oxidant state of the melanin polymer, thus possibly interfering with the digestion of cellular components including outer segment components [37].

Oxidative stress is recognized as a major pathogenic factor in age-related macular degeneration [38]. Diabetic conditions may lead to the accumulation of advanced glycation end products (AGEs) e.g. in the retinal pigment epithelium cell layers or photoreceptors [38, 39]. AGEs and also RAGE (receptor for AGEs) were also found in the retinal pigment epithelium or the retinal pigment epithelium and the photoreceptors in the maculae of human donor retina from patients with AMD, indicating to some extent similar biological pathways in AMD and diabetes [38, 40]. In the present study, antibodies against insulin were significantly upregulated in AMD patients.

Molecular chaperones and heat shock proteins are regulators for protein trafficking through cell organelle membranes and play a role in chaperoning damaged proteins to either the proteasome or lysosome for degredation [41, 42]. As stated above, autoantibodies against heat shock 70kDa protein 1A were significantly upregulated in the AMD 1 group in this investigation.

Logistic regression analysis showed, that the association between AMD and autoantibody level was still present after adjustment for smoking, body-mass-index, HbA1c level, level of high-density lipoproteins, of low-density lipoproteins and triglycerides in case of mitogen-activated protein kinase 3, lysozyme, transferrin and protein kinase C substrate 80K-H , insulin and heat shock 70 kDa protein 1A.

In general, it remains unclear whether the described autoantibodies play a causative role during the pathogenesis of AMD or appear as secondary effects during the disease’s progression. Several studies reported the involvement of antiretinal autoantibodies in age-related macular degeneration [10, 11, 43, 44]. Moreover, it has been suggested that certain autoantibody signatures may exist in different stages of age-related macular degeneration [11]. However, it still has to be elucidated whether the detected autoantibodies play a causative role in the pathogenesis of AMD or appear as secondary effects. Although the role of anti-retinal autoantibodies in the induction or progress of retinal degeneration presently is not well defined, it has been shown that anti-retinal autoantibodies can develop on average 3-15 years prior to the first clinical signs [45, 46]. In order to clarify the question whether the antiretinal autoantibodies play a causative role, future longitudinal studies, possibly with additional mass spectrometry to detect associated proteins, could be desirable.

Previous studies indicate that a causal association of autoantibodies with either pathogenesis or progression of AMD will require a higher number of subjects with early and late stages of AMD and that serum samples collected from the same patient, before and after AMD development or during progression of disease are not generally accessible [45]. A comparison of the retinal autoantibody profile in 63 individuals with and without early AMD from the Blue Mountains Eye Study showed an enhanced autoantibody response in early AMD [10]. In the GHS, the baseline examination with a total of 15,010 participants aged 35 to 74 years was conducted from 2007 to 2012. In this study, we evaluated data from a subsample of 1031 subjects enrolled in the GHS. In subsequent reports, after the 5- and 10-year follow-up of the same participants, the presence of antiretinal autoantibodies in early AMD stages might be associated with progression to later stages of AMD. Identification of serum autoantibodies associated with AMD might thus serve as a screening tool for prediction, give invaluable hints regarding prognosis as well as possibly monitor treatment response to intravitreal therapy in the future.

Strengths of the present study are the analysis by antigen-microarrays, an attractive tool which allows fast evaluation with high sensitivity of potential autoantibody biomarkers using microliter sample volumes. The present GHS study has a population-based, prospective design with broad assessment of phenotype information and large sample size. Fundus pictures of all participants and participants without AMD were graded independently by two experienced graders. This, to our knowledge, is the first report on microarray-based evaluation of autoantibody-levels in different AMD stages in a large German population-based cohort.

Since the GHS baseline recruitment was limited to participants below the age of 75, there were only 23 participants with AMD Stage 3 or 4, so these subjects had to be excluded for further statistical analysis. In addition, due to the relatively young age at the time of study entry, there were only 64 participants with AMD Stage 2 compared to 454 participants with AMD Stage 1 and 490 controls. The different group size explains the fact that significant differences in the antibody profile were only found between the controls and participants with AMD Stage 1. Subsequent reports will provide data on the autoantibody profile in different AMD stages compared to healthy controls after 5- and 10-year follow-up.

Any extrapolation to other populations should be done with caution, as the GHS cohort is predominantly Caucasian and of European ancestry. Although the age range reflects a broad proportion of the population, extrapolation of our data to other age groups may not be valid, especially as AMD is strongly associated with age.

## Conclusion

This study contributes towards a growing knowledge of autoantibodies in association with AMD by a microarray-based evaluation of human sera in the context of a large population-based, prospective, observational cohort study in Germany. Especially autoantibodies against inflammatory proteins were downregulated in participants with AMD.

### Supplementary information

ESM 1 (DOCX 17 kb)

## Data Availability

The analysis presents clinical data of a large-scale population-based cohort with ongoing follow-up examinations. This project constitutes a major scientific effort with high methodological standards and detailed guidelines for analysis and publication to ensure scientific analyses on highest level. Therefore, data are not made available for the scientific community outside the established and controlled workflows and algorithms. To meet the general idea of verification and reproducibility of scientific findings, we offer access to data at the local database in accordance with the ethics vote upon request at any time. The GHS steering committee, which comprises a member of each involved department and the head of the Gutenberg Health Study (PSW), convenes once a month. The steering committee decides on internal and external access of researchers and use of the data and biomaterials based on a research proposal to be supplied by the researcher. Interested researchers make their requests to the head of the Gutenberg Health Study (Philipp S. Wild; philipp.wild@unimedizin-mainz.de). More detailed contact information is available at the homepages of the GHS (www.gutenberghealthstudy.org) or the ophthalmic branch of the GHS (www.unimedizin-mainz.de/augenklinik/forschung/gutenberg-gesundheitsstudie.html).

## Abbreviations

AMD: age-related macular degeneration
GHS: Gutenberg Health Study
PBS: phosphate-buffered saline
MAPK: mitogen-activated protein kinase
ROS: reactive oxygen species
RPE: retinal pigment epithelium
Anti-CA II: anti-carbonic anhydrase II
CAR: cancer-associated retinopathy
AGE: advanced glycation end products
RAGE: receptor for advanced glycation end products
BMI: body mass index
Hba1c: glycated hemoglobin
HDL: high-density lipoproteins
LDL: low-density lipoproteins
OR: odds ratio
