# Supplementary material for: Association of autoantibody levels with different stages of age-related macular degeneration (AMD): Results from the population-based Gutenberg Health Study (GHS)
Source: Graefes Arch Clin Exp Ophthalmol. 2023 May 9;261(10):2763–73. doi: 10.1007/s00417-023-06085-2 (PMC10543519; doi:10.1007/s00417-023-06085-2)
Supplement: Supplementary file 1 — (DOCX 17 kb) [file 417_2023_6085_MOESM1_ESM.docx]

S1 Table : List of analysed antigens.

| Abbreviation | Full name | Supplier | Order number |
| --- | --- | --- | --- |
| ACO2 | Aconitate hydratase, mitochondrial | Abnova | H0000050-P01 |
| ACTA1 | Actin, alpha skeletal muscle | Sigma-Aldrich | A3653 |
| ALB | Serum albumin | Sigma-Aldrich | A9731 |
| ANXA5 | Annexin A5 | Sigma-Aldrich | A9460 |
| APOA1 | Apolipoprotein A-I | Antibody-Online | ABIN667126 |
| B_L_CRYS | β_L_-Crystallin | Sigma-Aldrich | C5163 |
| BDNF | Brain-derived neurotrophic factor | BioMol | 52615 |
| CA2 | Carbonic anhydrase 2 | Sigma-Aldrich | C6624 |
| CALR | Calreticulin | Sigma-Aldrich | C4714 |
| CKB | Creatine kinase B-type | Abnova | H00001152-P01 |
| CLUS | Clusterin | Enzo-LifeScienes | ALX-201-761-00500 |
| DCD | Dermicidin | Abcam | RPC896Hu01 |
| DPYSL2 | Dihydropyrimidinase-related protein 2 | Abnova | H00001808-P01 |
| ElF4A1 | Eukaryotic initiation factor 4A-I | Protagen |  |
| ENO2 | Gamma-enolase (NSE) | Sigma-Aldrich | N4773 |
| FN1 | Fibronectin | Sigma-Aldrich | F2006 |
| GAPDH | Glyceraldehyde-3-phosphate dehydrogenase | Abcam | ab77109 |
| GFAP | Glial fibrillary acidic protein | US Biological | G2032-33B |
| GLUL | Glutamine synthetase | Abnova | H00002752-P01 |
| GNB1 | Guanine nucleotide-binding protein G subunit beta-1 | Abnova | H00002782-P01 |
| GPD2 | Glycerol-3-phosphate dehydrogenase, mitochondrial | Protagen |  |
| GPX4 | Phospholipid hydroperoxide glutathione peroxidase, mitochondrial (GPX4) | Protagen | 159152842 |
| groEL2 | 60 kDa chaperonin 2 | Antibody-Online | ABIN1686691 |
| GST | Glutathione S-transferase | Abnova | P0001 |
| HARS | JO-1; Histidine--tRNA ligase, cytoplasmic | Sigma-Aldrich | J4144 |
| HSPA1A | Heat shock 70 kDa protein 1A | Sigma-Aldrich | H9776 |
| HSPB1 | Heat shock protein beta-1 | Enzo-LifeScienes | ADI-ESP-715-D |
| HSPD1 | 60 kDa heat shock protein, mitochondrial | Enzo-LifeScienes | adi-nsp-540 |
| HSPE1 | 10 kDa heat shock protein, mitochondrial | US Biological | C3450 |
| IGLL1 | Immunoglobulin lambda-like polypeptide 1 | Abcam | ab140547 |
| INS | Insulin | Sigma-Aldrich | I6634 |
| LPPR3 | Phospholipid phosphatase-related protein type 3 | Protagen |  |
| LYZ | Lysozyme C | Sigma-Aldrich | L6876 |
| MAPK3 | Mitogen-activated protein kinase 3 | Protagen |  |
| MBP | Myelin basic protein | Sigma-Aldrich | M1891 |
| MUC5B | Mucin-5B | Abcam | RPA684HU01 |
| NTF3 | Neurotrophin-3 | R&D Systems | 267-N3/CF |
| NTF4 | Neurotrophin-4 | R&D Systems | 268-N4/CF |
| OGFR | Opioid growth factor receptor | Protagen |  |
| PDIA3 | Protein disulfide-isomerase A3 | Abcam | ab92937 |
| PEBP1 | Phosphatidylethanolamine-binding protein 1 | Abnova | H00005037-P01 |
| PKC | Protein kinase C | Sigma-Aldrich | P3115 |
| PPIA | Peptidyl-prolyl cis-trans isomerase A | Sigma-Aldrich | C3805 |
| PRKCSH | Glucosidase 2 subunit beta | Protagen |  |
| SCFD1 | Sec1 family domain-containing protein 1 (VTI-B) | Protagen |  |
| SERPINA | Alpha-1-antitrypsin | Sigma-Aldrich | A9024 |
| SFN | 14-3-3 protein sigma | Enzo-LifeScienes | BMLSE488 |
| SNCA | Alpha-synuclein | Protagen |  |
| SNCG | Gamma-synuclein | Sigma-Aldrich | S3071 |
| SOD | Superoxide dismutase | Sigma-Aldrich | S7446 |
| SPTA1 | Spectrin | Sigma-Aldrich | S3644 |
| SRP14 | Signal recognition particle 14 kDa protein | Protagen |  |
| TF | Serotransferrin | Sigma-Aldrich | T3309 |
| TG | Thyroglobulin | Sigma-Aldrich | T1001 |
| TNF | Tumor necrosis factor | Milipore | GF026 |
| TNNI3 | Troponin I, cardiac muscle | Abcam | RPA478HU01 |
| TOP1 | DNA topoisomerase 1 | US Biological | T8065-02 |
| TTR | Transthyretin | Sigma-Aldrich | P1742 |
| UCHL1 | Ubiquitin carboxyl-terminal hydrolase isozyme L1 | Abnova | H00007345-P01 |
| USP10 | Ubiquitin | Sigma-Aldrich | U5507 |
| VEGF | Vascular endothelial growth factor | Sigma-Aldrich | V7259 |
